# Polarimetric-Based Analysis and Manufacturing of Dye-Doped Liquid Crystal Photoaligned Cells for the Visible Range

**DOI:** 10.3390/polym17182489

**Published:** 2025-09-15

**Authors:** Adrián Moya, Adriana R. Sánchez-Montes, Emilio J. Mena, Manuel Ortuño, Mariela L. Álvarez, Eva M. Calzado, Andrés Márquez

**Affiliations:** 1 Instituto Universitario de Física Aplicada a las Ciencias y las Tecnologías, Universidad de Alicante, E-03080 Alicante, Spain; adrianaros.sanchez@gcloud.ua.es (A.R.S.-M.); emilio.mena@mscloud.ua.es (E.J.M.); mos@ua.es (M.O.); mariela.alvarez@gcloud.ua.es (M.L.Á.); evace@ua.es (E.M.C.); andres.marquez@gcloud.ua.es (A.M.); 2 Departamento de Física, Ingeniería de Sistemas y Teoría de la Señal, Universidad de Alicante, E-03080 Alicante, Spain

**Keywords:** liquid crystal, photoalignment, methyl red, thickness, diattenuation, retardance

## Abstract

The accurate and controlled alignment of liquid crystals (LCs) in modern optical devices is of great importance. Photoalignment is one of the most appealing approaches for achieving more versatile alignment in designs. One of the most important parameters of these devices is the thickness and the homogeneity in the photoaligned area, especially in devices that introduce retardance. In this work, we propose a novel polarimetric-based method for the measurement of thickness of homogeneous liquid crystal cells that considers diattenuation effects and how they affect the retardance generated by a liquid crystal variable retarder (LCVR). We experimentally demonstrate the production of dye-doped liquid crystal (DDLC) devices, photoaligned in the visible range with a 532 nm laser light, of two different thicknesses with a very high spatial homogeneity. Thinner devices can be used across the whole visible spectrum despite the residual diattenuation at shorter wavelengths, whereas thicker ones achieve the best degree of polarization (DOP) in the transmitted wavefronts, close to 100%, at longer wavelengths.

## 1. Introduction

In the fabrication of liquid crystal (LC) optical devices, molecular alignment is critical to device performance and light interaction. While traditional methods, like mechanical rubbing [1,2], microgroove surfaces [3], laser patterning [4], among other methods [5], are widely used, they often involve irreversible modifications to the substrate, which can be problematic, especially for complex surfaces. Photoalignment offers a non-contact alternative method using light and photosensitive materials, enabling flexible surface compatibility and the creation of multi-domain alignments within a single cell [6,7,8,9]. In addition, this technique is very versatile, since it can be used in all kinds of LCs and other materials such as gel networks, conjugated polymers, organic semiconductors, among others [10].

Among the various photoalignment materials, azo dyes stand out for their ability to reorient in response to polarized light through cis-trans isomerization [11,12,13]. Notably, Methyl Red (MR) is distinguished by its solubility in liquid crystals, which enables the formation of dye-doped liquid crystals (DDLCs) [14,15,16]. When exposed to green light, MR molecules undergo Brownian diffusion within the cell and become adsorbed onto the substrates via electrostatic and dipole interactions. Through repeated isomerization and adsorption cycles, MR molecules align perpendicular to the light’s polarization, ultimately inducing a parallel alignment of the surrounding LC molecules, making it a powerful tool for achieving efficient photoalignment without physically modifying the substrate [12,17].

Most photosensitive materials used in photoalignment techniques typically absorb in the ultraviolet UV region of the spectrum. As a result, working with these materials requires specialized optical components designed for UV light, which are not as widespread as the ones for the visible range. This complicates the experimental set-up, increases costs, and limits access to the fabrication of photoaligned LC photonic devices to most laboratories which are not specialized in the specifics of organic chemistry and LC materials. In this paper, we explore the development of “all-with-visible light” LC devices. In doing so, we aim to address the main limitation (diattenuation) associated with the fabrication and practical use of such devices within this spectral range. In this sense, Methyl Red is a very appealing material since it absorbs across almost the entire visible spectrum, except in the red region [18,19]. As a result, optical effects such as diattenuation are expected to significantly influence the phase retardance, making the devices practically unfeasible to use over a broad visible range. Nevertheless, the impact of diattenuation in MR-based devices remains largely unexplored, despite its critical influence on their optical performance.

For the optimal performance of the manufactured devices, a detailed analysis of the thickness and spatial homogeneity is necessary. Currently, a wide range of methods are available for measuring the thickness of LC devices and other LC parameters. Among the most used techniques are the wave retarder rotational method [20], the phase compensation method [21], the snapshot measurement [22], and heterodyne interferometry [23]. However, when the thickness is the only parameter of interest, the most used approach is the spectral analysis of the light transmitted through an empty cell, due to its simplicity and the speed with which accurate results can be obtained [24,25]. Despite the extensive literature on thickness measurements, very few studies have focused on photoaligned LC devices. Azo dyes are photosensitive materials and therefore might exhibit diattenuation effects, as previously discussed. However, there are very few methods that study this parameter. These two parameters, diattenuation and thickness, are of great importance because they both greatly affect the retardance induced by the LC devices. In the paper, we show that a polarimetric-based approach is especially suited for their characterization and analysis. This is something that we have exploited with other LC devices, such as the liquid crystal on silicon (LCoS) spatial light modulators (SLMs) [26,27], where polarimetric approaches are more effective in revealing in-depth information when compared with diffractive and interferometric approaches [28].

In this work, we present a polarimetric-based methodology for applying the photoalignment technique to fabricate liquid crystal variable retarders (LCVRs) with a homogeneous distribution of the LC molecules, which we will refer to as uniform alignment for the rest of the paper. We demonstrate that this approach enables us to determine the cell thickness with high precision after the device has been fully assembled and is operational, as well as to assess its degree of spatial homogeneity. To achieve this goal, we develop a theoretical framework that combines both linear retardance and diattenuation in LCVRs and assess its impact on the resulting polarization states across the visible spectrum. The results obtained demonstrate that these devices can also be used in a wider visible spectral range beyond the red region.

The structure of the paper is as follows. First, in Section 2, we will present the theoretical development of our methodology. In Section 3, we present the manufacturing of the devices and the experimental set-up. Finally, in Section 4, we will show and discuss the results.

## 2. Thickness Methodology of Filled LC Cells

The most basic property we deal with for LC-based devices is probably the retardance (Γ). This is equal to the delay experienced by the orthogonal components of the electric field as they oscillate along the ordinary and extraordinary axis (also called LC director axis), given by

(1) $$\Gamma =\Delta \varphi =\varphi_{e}-\varphi_{o}=\frac{2\pi \cdot \Delta n(\lambda ,\alpha )\cdot d}{\lambda }$$

where $\Delta n\left(\lambda ,\alpha \right)=n_{e}\left(\lambda ,\;\alpha \right)-n_{o}\left(\lambda \right)$ is the difference between the extraordinary ($n_{e}$) and ordinary ($n_{o}$) refractive indexes, α is the rotation angle (or tilt angle) of the molecules relative to their rest position, which normally, in nematic LC, consists of the extraordinary axis along the substrate, and d is the thickness of the device [29].

Equation (1) provides a range of options for the characterization of LC devices, including the calculation of the thickness, which is normally an unknown parameter unless you are manufacturing the device. To perform this measurement, it is essential to consider that when measuring the phase difference between the orthogonal components of the electric field, the wrapped phase is typically obtained. Mathematically, the absolute retardance Γ is equivalent to the wrapped retardance $\Gamma_{w}$ plus m times 2π, where m is a positive integer expressing the multiplicity, $\Gamma =m\cdot 2\pi +\Gamma_{w}$. In general, a measurement device like a polarimeter measures the wrapped retardance $\Gamma_{w}$. So to obtain the absolute retardance and the thickness of the device, it is necessary to calculate the different multiplicities $m_{\lambda_{i}}$.

One way of obtaining the value of the unwrapped retardance is to measure the wrapped one ($\Gamma_{w,\;\lambda }$) at a chosen point on the device for two different wavelengths. Then, looking at Equation (1), and taking the quotient between the measurements at two wavelengths $\lambda_{1}$ and $\lambda_{2}$, we obtain the following relation:

(2) $$\frac{\Gamma_{{w,\lambda }_{1}}+2\pi m_{\lambda_{1}}}{\Gamma_{{w,\lambda }_{2}}+2\pi m_{\lambda_{2}}}=\frac{\Delta n\left(\lambda_{1}\right)}{\Delta n\left(\lambda_{2}\right)}\frac{\lambda_{2}}{\lambda_{1}}$$

The right side of Equation (2) is easy to obtain, as λ is known and Δn(λ) can be obtained from the literature [30]. In Table 1, we show the values for Δn for the nematic LC E7 for three wavelengths, sampling the whole visible spectrum.

Then, the unknown variables are on the left side of Equation (2), where the wrapped retardance $\Gamma_{w,\lambda }$ can be obtained from polarimetric measurements, as we will show afterwards. Then, the only unknown variables left are the multiplicities $m_{\lambda_{i}}$. Since the value for the quotient on the right side of Equation (2) is known, these $m_{\lambda_{i}}$ can be calculated giving arbitrary values to them until both sides of the equation are equal. Note that the value of the $m_{\lambda_{i}}$ is unique for each of the wavelengths $\lambda_{i}$.

It was mentioned that the wrapped retardance can be measured using a polarimeter. This measurement is not direct, but it can be deduced from ellipticity measurements. For this goal, we use the Mueller–Stokes formalism to develop a model for a polarization device exhibiting linear retardance and diattenuation. To express the different vectorial magnitudes along the paper, we will consider a right-handed reference system, where the *x* and *y* axes are along the horizontal and vertical directions, respectively, with the *z*-axis pointing along the light propagation direction, and where right (left) handedness corresponds to clockwise (counterclockwise) sense of direction from where the light is coming. This coincides with the reference system of the polarimeter used in this work. We will show that through polarimeter measurements, we can provide a methodological evaluation of parameters relevant for the manufacturing process of the photoaligned DDLC devices, such as the thickness.

It is known that the ellipticity angle (χ) is related to the Stokes parameters $S_{i}$ in the following way [31]:

(3) $$\sin2\chi =\frac{S_{3}}{S_{0}}$$

In our approach, we can directly connect the ellipticity χ with the wrapped retardance $\Gamma_{w,\;\lambda }$, and for that we, need to obtain the appropriate mathematical relation. In the case of this paper, as it will be shown below, the device is a LC variable linear retarder (LCVR) with linear diattenuation, i.e., it behaves simultaneously as a retarder and a polarizer, depending on the incident wavelength. The components of the transmitted electric field can then be expressed in the orthogonal neutral lines of the device as follows:

(4) $${E^{\prime }}_{x}=p_{x}e^{i\frac{\Gamma }{2}}E_{x}$$

(5) $${E^{\prime }}_{y}=p_{y}e^{-i\frac{\Gamma }{2}}E_{y}$$

where $p_{x}$, $p_{y}$ are the attenuation coefficients of the field amplitude, Γ is the linear retardance induced by the retarder behaviour, and E and $E^{\prime }$ are the electric field before and after going through the device, respectively.

The Stokes parameters of the output and input beam can be calculated as follows [31]:

(6) $${S^{\prime }}_{0}={E^{\prime }}_{x}\cdot {{E^{\prime }}^{*}}_{x}+{E^{\prime }}_{y}\cdot {{E^{\prime }}^{*}}_{y}$$

(7) $${S^{\prime }}_{1}={E^{\prime }}_{x}\cdot {{E^{\prime }}^{*}}_{x}-{E^{\prime }}_{y}\cdot {{E\prime }^{*}}_{y}$$

(8) $${S^{\prime }}_{2}={E^{\prime }}_{x}\cdot {{E^{\prime }}^{*}}_{y}+{E^{\prime }}_{y}\cdot {{E\prime }^{*}}_{x}$$

(9) $${S\prime }_{3}={i(E^{\prime }}_{x}\cdot {{E^{\prime }}^{*}}_{y}-{E^{\prime }}_{y}\cdot {{E^{\prime }}^{*}}_{x})$$

and since the Stoke vector of the output beam *S*’ is given by

(10) $$S^{\prime }=M(p_{x},p_{y},\Gamma )\cdot S$$

we can deduce the Mueller matrix $M\left(p_{x},p_{y},\Gamma \right)$ of the device in its neutral lines:

(11) $$M\left(p_{x},p_{y},\Gamma \right)=\left(\begin{array}{cccc} \frac{p_{x}^{2}+p_{y}^{2}}{2} & \frac{p_{x}^{2}-p_{y}^{2}}{2} & 0 & 0\\ \frac{p_{x}^{2}-p_{y}^{2}}{2} & \frac{p_{x}^{2}+p_{y}^{2}}{2} & 0 & 0\\ 0 & 0 & p_{x}p_{y}cos\Gamma & p_{x}p_{y}sin\Gamma \\ 0 & 0 & {-p}_{x}p_{y}sin\Gamma & p_{x}p_{y}cos\Gamma \end{array}\right)$$

If we consider that the incident state of polarization (SoP) corresponds to linearly polarized light oriented at +45°, i.e., the normalized input Stokes vector *S* is given by *S* = (1, 0, 1, 0), then the output SoP, *S*’, is expressed as

(12) $$S^{\prime }\left(\Gamma ,45^{o}\right)=\frac{1}{2}\left(\begin{array}{c} p_{x}^{2}+p_{y}^{2}\\ \begin{array}{c} p_{x}^{2}-p_{y}^{2}\\ \begin{array}{c} 2p_{x}p_{y}\cos\Gamma \\ -2p_{x}p_{y}sin\Gamma \end{array} \end{array} \end{array}\right)$$

and by applying Equation (3) to the previous expression, we determine that the retardance (wrapped values $\Gamma_{w}$) is modulated by the diattenuation of the device as follows:

(13) $$sin2\chi =\frac{-2p_{x}p_{y}}{\left(p_{x}^{2}+p_{y}^{2}\right)}sin\Gamma_{w}$$

Note that Equation (13) is not capable of providing the absolute retardance due to the periodicity of the “sin” functions. As a final check, if it is considered that the device is a simple retarder without diattenuation, $p_{x}=p_{y}=p$, then the relation is the same as the one typically found in bibliography, $\sin2\chi =-\sin\Gamma_{w}$ [31]. An alternative way to obtain the retardance $\Gamma_{w}$ is given through the quotient −S3S2 in Equation (12), that is

(14) Γw=atan−S3S2

However, in this paper, we find it more convenient to use Equation (13), as we will show when discussing the effects of diattenuation in LCVRs in Section 4.2, since this expression contains all the relevant information.

In order to accurately measure the absorption parameters, it is necessary to confirm that the device’s neutral lines are orthogonal and aligned along the *x*-axis (laboratory’s horizontal) and *y*-axis of our reference system. To verify this, the manufactured device is placed between crossed linear polarizers, and we checked that there is no light leakage along these perpendicular orientations, confirming that our device has a uniform alignment, where the LC molecules have their long (LC-director orientation) and short axis along the *x*-axis and *y*-axis, respectively. Finally, the absorption factors can be measured by placing a linear polarizer in front of the device, with its transmission axis along the neutral lines of the device. The resultant power of the light after going through the device is captured by a radiometer for three different wavelengths (473 nm, 532 nm, and 633 nm), sampling the visible spectrum. Alternatively, if instead of the ellipticity, we measure the Stokes parameters for the light at the output of the sample, we can extract $p_{x}$ and $p_{y}$ from Equation (12) as follows:

(15) $$p_{x}=\sqrt{S_{0}+S_{1}}$$

(16) $$p_{y}=\sqrt{S_{0}-S_{1}}$$

Knowing the wrapped retardance, the refraction indexes Δn, the wavelengths $\lambda_{i}$, and the multiplicities $m_{\lambda_{i}}$, using Equation (2), the absolute retardance Γ can be obtained, and using Equation (1), the thickness can be calculated.

## 3. Experimental Methods

### 3.1. Materials

Photoaligned devices based-on Methyl Red-doped liquid crystals with different thicknesses were fabricated. For this purpose, 25 mm × 37.5 mm glasses with a 23 nm thick Indium Tin Oxide (ITO) layer were used as substrates. The initial stage in the fabrication of liquid crystal devices is the surface treatment. The substrates were subjected to a cleaning process involving acetone (Merck, Darmstadt, Germany) and an ultrasonic bath (Equipos Clínicos, Jaén, Spain) for a duration of 15 min followed by drying in an oven (fisher scientific, Madrid, Spain) at a temperature of 120 °C for 20 min. Lastly, the surfaces were treated with an Ossila UV Ozone Cleaner (Ossila, Sheffield, UK) for 15 min and after that, the cells were assembled with two substrates, with the thickness of the cell being defined by the silica spacers (5.5 μm and 10 μm; Whitehouse Scientific, Chester, UK). The size was carefully considered, so that retardances larger than 2π are achievable, taking into account Equation (1) and the birefringence values shown in Table 1. The spacers were deposited using the spin-coating technique, and the two sides of the cell were sealed with UV adhesive (Norland Optical Adhesive 61, Edmund Optics, Barrington, NJ, USA).

A mixture consisting of 99 wt% LC E7 (Nematel, Mainz, Germany) and 1 wt% Methyl Red MR (Merck, Darmstadt, Germany) was introduced by capillary action inside the cells. To facilitate the capillary process and ensure a uniform distribution of the LC between substrates, it was essential to maintain a temperature of approximately 65 °C, at which point the LC is in an isotropic phase, meaning that the molecules behave like a liquid and consequently lose their birefringence. Thanks to this, it is feasible to photoalign both sides of the device simultaneously. This is called double side photoalignment [32]. Finally, for the device of 5.5 μm, a power density of 600 mW/cm^2^ for 60 min was needed for correct photoalignment, while for the 10.0 μm device, a power density of 90 mW/cm^2^ for 85 min was used.

### 3.2. Experimental Set-Up

In our study, it is very important to know the thickness of our samples. For this reason, the thickness of empty cells was measured using a simple set-up consisting of a supercontinuum laser combined with a wavelength selector (SuperK Varia, NKT Photonics, Birkerød, Denmark) and a spectrometer (Black Comet, Stellar Net, FL, USA). The cells were illuminated by the laser operating at its maximum spectral bandwidth covering the range 400–900 nm. The beam passing through the cell was collected with an optical fibre and the transmission spectrum was analyzed in a spectrometer to obtain the thickness of the device. To measure the diattenuation parameter, the supercontinuum laser is used again, but only for three wavelengths, 473 nm, 532 nm, and 633 nm, with a selected spectral bandwidth of 10 nm.

**Figure 1:**
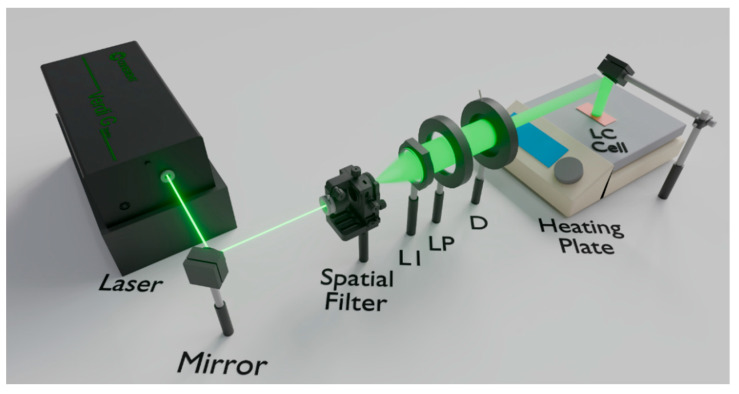
Set-up for photoaligning MR cells (L1: Lens; LP: Linear Polarizer; D: Diaphragm).

The photoalignment set-up is illustrated in Figure 1. First, the 532 nm laser beam (Verdi-V5, Coherent, Copenhagen, Denmark) is expanded by a spatial filter, after which it passes through a lens (L1) to collimate it and a linear polarizer (LP1) that generates linearly polarized light along the vertical axis of the laboratory. Finally, with a diaphragm (D), a circular area with a diameter of 1 cm was obtained.

During the whole process, the liquid crystal must be kept in an isotropic state to ensure a correct homogeneous photoalignment area on the samples. Otherwise, the birefringent behaviour of the LC molecules would cause the polarization that reaches each substrate to be different. This way, the LC molecules align themselves with their long axis along the horizontal of the laboratory.

Eventually, to characterize the devices fabricated and their performance as a LCVR, all of them were illuminated with a linear polarized beam at 45° and wavelength of 633 nm (He-Ne laser, Lasing SA, Madrid, Spain). The beam passing through the cell was collected by a polarimeter (PAX5710VIS_T, Thorlabs, NJ, USA), which allows us to obtain information about the light through the devices in terms of azimuth, ellipticity, degree of polarization (DOP), and power.

## 4. Results: Cell Parameter Evaluation and LCVR Performance

Using the experimental set-ups presented in the previous section, all devices were characterized by studying the thickness, the diattenuation, and the polarimetric behaviour as a LCVR.

### 4.1. Cells Thickness

As mentioned in Section 3.1, devices with commercial spacers of 5.5 and 10 μm had been fabricated. It is important to check the thickness of the devices as the retardance is a very sensitive parameter to changes in the cell gap. Therefore, using the experimental set-up explained in the last section, the thickness can be measured.

**Figure 2:**
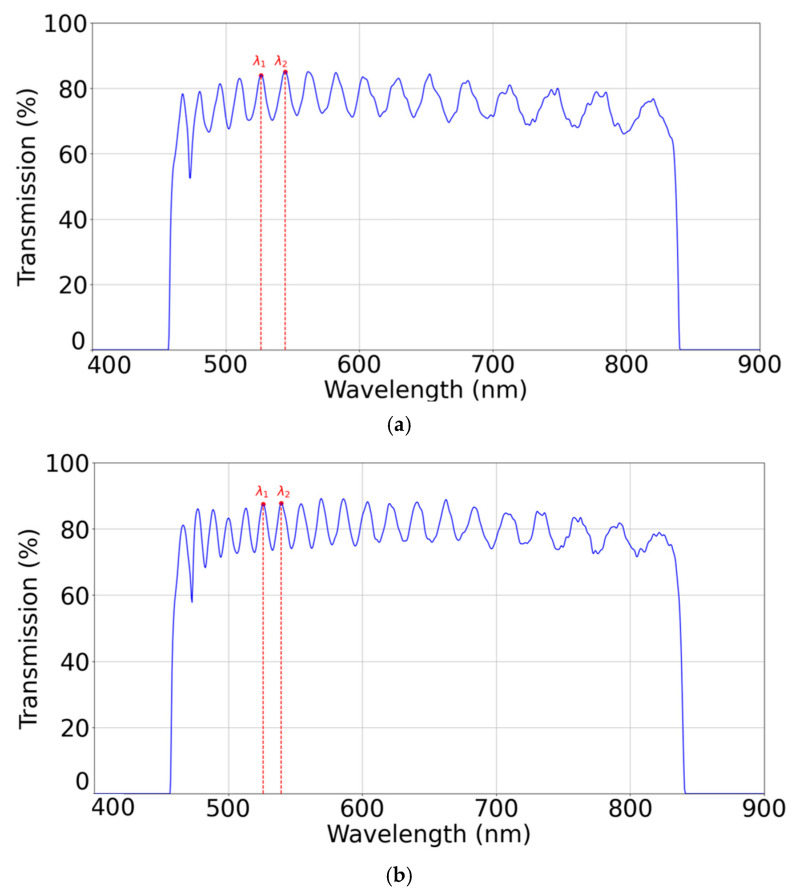
Transmission spectrum of empty cell of (**a**) 5.5 and (**b**) 10 μm in its central point. Two wavelengths used in Equation (17) are marked.

On the one hand, this interferometric-based method enables the measurement of the cell’s thickness before it is filled with LCs. When an empty cell is illuminated by a coherent light beam perpendicular to the device, multiple reflections are produced at the air–glass interface. The reflections and transmissions are also perpendicular to the substrates, resulting in the superposition of several interferences. The measurement detected by the spectrometer is a transmission spectrum with a series of spectral ripples, well known to be related to Fabry–Pérot effects, as shown in Figure 2. The distance between spectral peaks gives us information about the thickness of the cell, given by the following expression [22]:

(17) $$d=\frac{\lambda_{1}\lambda_{2}N}{2\left(\lambda_{2}-\lambda_{1}\right)}$$

where *N* is the number of peaks between $\lambda_{1}$ and $\lambda_{2}$. This is carried out for five points across the aperture of the device as we will explain with more detail afterwards. As an example, in Figure 2, we show the measurement for the central point of the devices at each of the two thicknesses.

On the other hand, by applying the theoretical concepts outlined in the Introduction to the proposed polarimetric-based approach, the thickness of the device can be deduced. A distinguishing feature of this more general approach is that the sample is already filled with LC and photoaligned. In the following, we consider its extraordinary axis (LC-director) oriented along the horizontal of the laboratory (*x*-axis in the laboratory reference system). Hence, by transmitting linearly polarized light at 45° to the device and collecting it with a polarimeter, Equation (13) can be employed to calculate the wrapped retardance, and Equation (1) for the thickness, thus allowing for comparison with the result obtained by the spectrometer. Since the photoaligned area is 1 cm in diameter, five measurements distributed along the photoaligned area were conducted to obtain the average value of the cell thickness. These points are equidistant from each other, with two points situated to the left and right from the central point at a distance of 3 mm, and an additional two points located at the top and bottom at the same distance to the central point.

Following the explanation at the end of Section 2, in Table 2, the absorption factors were measured for two areas of the material, one with photoalignment and the other without it (non-illuminated). We are measuring five points in our device to check the homogeneity within the photoaligned area and also in the non-photoaligned. In the case of the non-illuminated area, we measured five points taken around the photoaligned area at a distance of 15 mm to the centre of the sample. In Table 2, we show the average values together with the typical deviation (root mean square difference with respect the average value).

These results are consistent with the absorption spectrum of Methyl Red MR [17], where the absorption in red (633 nm) is very low, while it is more significant in green (532 nm) and blue (473 nm). There is also, as expected, more absorption along the director axis (*x*-axis) due to the way the MR molecules have aligned.

**Figure 3:**
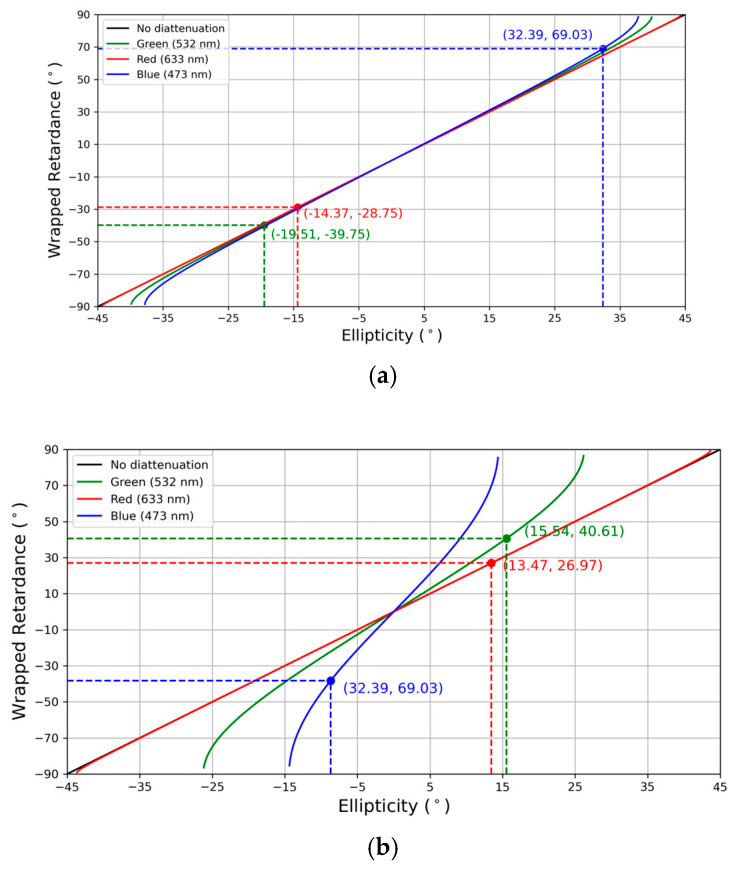
Relation between ellipticity and retardance for thickness cells of (**a**) 5.5 and (**b**) 10 μm.

Using Equation (13) and the measured amplitude absorption parameters, we plot in Figure 3 the wrapped retardance as a function of the ellipticity, both for the thinner and the thicker samples, respectively, in (a) and (b). In each of the figures, as expressed in the legend, we show how the diattenuation affects the determination of the retardance of the devices in comparison to the ideal case without diattenuation.

Following the theoretical method, at the same five points where the absorption parameters were measured and with an incoming 45° linear polarized light, the ellipticity was recorded and the wrapped retardance was calculated for three different wavelengths using Equation (13). The average values of the ellipticity and the resulting wrapped retardances are presented in Figure 3.

As it is shown in Figure 3, for the 5.5 μm thickness, the diattenuation has little effect on the $\Gamma_{w}$ of the device; there are some light variations in green and blue, while red is almost overlapped with the no-diattenuation line. At 10 μm, however, it produces significant changes that must be considered when characterizing the devices. As expected, in the same way as the thinner device, red does not experience considerable modification in the retardance because the absorption is almost zero. An interesting phenomenon that can be observed, especially in the 10 μm device, is that there is a constraint on the achievable ellipticities for different wavelengths that will be further analyzed in the next section.

With all these data, we can obtain the average thicknesses from the five sampled points within the 1 cm diameter illuminated area. This is performed for the two methods described and for the two devices together with the associated typical deviation, and it is shown in Table 3.

As demonstrated in Table 3, the real thicknesses of the manufactured devices exceed the dimensions of the spacers. However, both methods yield highly comparable results, thereby validating the thicknesses as accurate. From now on, when discussing the LC devices, the average of their actual thicknesses, 8.4 and 12.0 μm, will be employed. We also observe that we can achieve an almost constant thickness across the 1 cm aperture, which is crucial for certain applications.

### 4.2. Polarimetric Characterization of Uniformly Aligned Cells

As has been shown throughout this Results Section, achieving a spatially uniform alignment of the liquid crystal is highly beneficial for evaluating the fabrication process of the cells and it enables a straightforward interpretation of the results. In this last section, taking advantage of the uniform LC distribution, we will investigate the behaviour of our devices under an applied voltage, in which case they function as liquid crystal variable retarders (LCVRs).

To establish a robust definition of a successful photoalignment, it is necessary to concentrate on two optical parameters: the azimuth of the output light (orientation angle of the polarization state) and the degree of polarization (DOP). The azimuth, DOP, and the capacity of this device to generate multiple states of polarization at the output, including linear, elliptical, and circular polarized light, by modulating the retardance through the variation in the applied voltage were studied. As already mentioned, these devices are LCVRs with their LC director, that is the alignment direction, along the horizontal of the laboratory (*x*-axis). Thus, if 633 nm light linearly polarized with DOP close to 100% and with an azimuth of 45° to the horizontal is used, ideally, the output beam will be elliptically polarized, with an azimuth of 45° and the same DOP as the input beam. Therefore, a sample is considered to be well photoaligned when the azimuth is close to 45° and the DOP is higher than 90%.

This study was carried out for the two device thicknesses discussed within the paper. In Table 4, we show, for 0 V, the azimuth, the ellipticity, and the DOP of five points distributed within the photoaligned area (as described in Section 4.1), where the ellipticity values are the ones used in Section 4.1 to obtain the thickness in the polarimetric approach. As demonstrated in this table, the sample with a thickness of 8.4 μm exhibits notable spatial homogeneity, with an almost constant azimuth and ellipticity across all the five points. Furthermore, the DOP is consistently above 90% in all cases.

**Figure 4:**
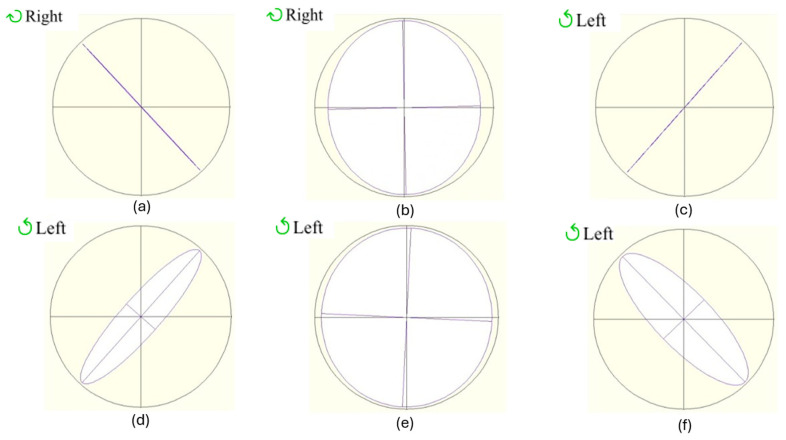
The 8.4 μm thickness device voltage study at the point in the centre: (**a**) 1.1 V; (**b**) 2.1 V; (**c**) 2.6 V; (**d**) 3.0 V; (**e**) 4.3 V; (**f**) 10.0 V.

Next, the central point was chosen to carry out a sweep of voltages to see how the retardance varies and, therefore, the polarization states that the device can generate. As it is shown in Figure 4 and Table 5, the presence of well-defined linear, elliptical, and circular light is evident, with azimuths approaching 45° (excluding the circular pattern, where the azimuth angle is not defined) and DOP values exceeding 90%. All these figures are obtained from the polarimeter in the same reference system used throughout the paper.

The higher the voltage, the better the performance of the device, with azimuths closer to 45° and higher DOPs.

**Figure 5:**
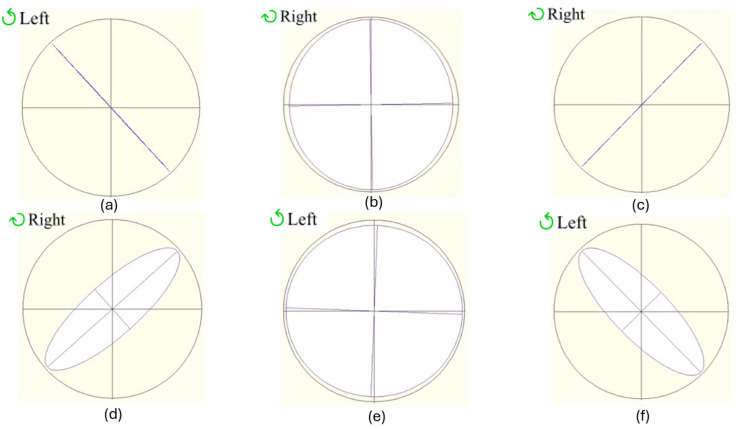
The 12.0 μm thickness device voltage study at the point in the centre: (**a**) 1.1 V; (**b**) 1.5 V; (**c**) 1.7 V; (**d**) 3.1 V; (**e**) 6.5 V; (**f**) 16.0 V.

The same study just shown for the thinner cell was repeated for the 12.0 μm thickness device. In Table 6, the spatial homogeneity of the device is demonstrated, and in Figure 5 and Table 7, the polarimetric behaviour is shown (with the ellipticity values used for the thickness calculation). The performance of the thicker device at 633 nm is comparable to and, in certain instances, superior to that of the 8.4-micron device. This comparison is further demonstrated in Table 6 and Table 7, which illustrate that the trend of the behaviour is consistent with the previous case, yet the spatial homogeneity of the device, the azimuth (proximity to 45°), and the DOP (proximity to 100%) are significantly enhanced.

It is important to note that high quality circularly polarized light is possible with ellipticity and DOP values around 45° and 100%, respectively, as shown quantitatively in Table 7. This is a very relevant result demonstrating the optimal approach for the manufacturing and the analysis undertaken in the present paper.

**Figure 6:**
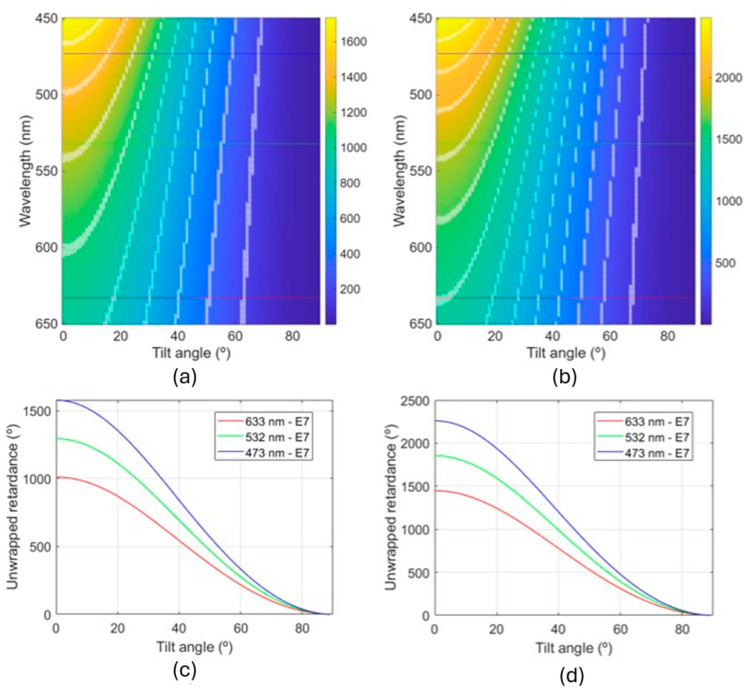
Colour plots of the retardance vs. tilt angle for the visible spectrum for the (**a**) 8.4 μm and (**b**) 12.0 μm devices, where each overlapped white line is a π rad multiple of the retardance. Axial cut for the three wavelengths used in the paper to sample the visible spectrum for the (**c**) 8.4 μm and (**d**) 12.0 μm devices.

To provide further insight into the tunability of the retardance with the applied voltage, in Figure 6, it is shown how the retardance varies as a function of the wavelength and tilt angle for E7 (the LC used in this work) in a 2D colour plot for the 8.4 μm (Figure 6a) and 12.0 μm (Figure 6b) devices, respectively. In Figure 6c,d, an axial cut of Figure 6a,b is shown at the three sampled wavelengths in this work, where the evolution of the retardance with the tilt angle can be more clearly seen, and the higher the tilt angle, the lower the rate of variation in the retardance. It is important to consider that the relationship between the applied voltage and the tilt angle (angle between the long axis of the LC molecule and the plane of the substrate) is not linear. However, it is established that at a voltage of 0 V, the tilt angle is 0°, and at large voltages, the tilt angle approaches 90°. It can be seen, especially in Figure 6c,d, how the retardance changes fast at low voltages and shows more stable behaviour at higher voltages. Moreover, the graphs provide insight into the range of retardance that the devices can introduce to the incoming light, and how, following Equation (1), the increase in thickness implies an increase in the retardance generated.

An additional verification procedure can be performed using Figure 6a,b and the number of retardance multiples of π are detected during the voltage sweep. Every time we have linear light with azimuths of 45° and −45°, the retardance difference between them is π rad. As illustrated in Figure 6a for a wavelength of 633 nm, the maximum observable multiple of π rad is five. However, experimental results demonstrate the observation of four multiples of π, which is a highly satisfactory outcome given the typically limited access to the complete effective range of delays, due to the necessity of extremely high voltages to completely tilt the LC molecules. In comparison, the thicker device of Figure 6b shows seven multiples of π rad, i.e., all those predicted by the simulation except the last one, happening at the larger tilt angle, with the same behaviour as the thinner device. So, the 8.4 μm device can generate a retardance of 4π rad and the 12.0 μm device a retardance of 7π rad at 633 nm. In optical applications, a range of 2π rad is generally enough. In the case of our devices, they can generate this range of retardance for a voltage interval of 1.0 V–1.7 V and 1.1 V–1.5 V, for the 8.4 μm and 12.0 μm devices, respectively, by applying very low voltages, which is particularly interesting in practical applications.

As previously mentioned in the experimental set-up section, the LCVR behaviour of the devices was characterized using only the 633 nm wavelength, where MR exhibits almost no absorption. However, revisiting the results from Figure 6, it is worth noting that the 12.0 μm device (Figure 6b) cannot be used effectively as a LCVR at the other two wavelengths (473 nm and 532 nm) because, as previously mentioned, the ellipticity range is between −26° and 26° for 532 nm and between −14° and 14° for 473 nm, so many polarization states cannot be generated, including circular light. In contrast, the 8.4 μm device shows a much smaller retardance variation at those two wavelengths. Although it still does not cover the full 45° range, it enables access to most polarization states, including a quasi-circular polarization state with ellipticities of approximately 40° and 38° for the 532 nm and 473 nm wavelengths, respectively. With these results, we can deduce that thinner devices are relatively unaffected by diattenuation, allowing them to still be used in the visible spectrum. It is expected that the thinner the device is, the lower the impact of the diattenuation. This is a significant outcome of this work since this means that “all-with-visible light” devices are possible. Fabrication (photoalignment) can be performed using the typical infrastructure of lasers and optical equipment found in many laboratories, without compromising the performance when being afterwards illuminated with the visible spectrum in applications.

## 5. Conclusions

In this paper, a methodology for the optimal fabrication of liquid crystal variable retarders (LCVRs) using the azo dye photoalignment technique is developed. In the context of azo dyes, which absorb within the visible spectrum, the phenomenon of diattenuation appears. This issue limits the functionality of the manufactured devices along the shorter wavelength range in the green and blue parts of the visible spectrum. The proposed method facilitates the characterization of this diattenuation and how it affects the devices, and it will be very useful for our future research. This also allows for an alternative means of determining important device parameters, such as retardance and thickness, once the device is already mounted and operational. Furthermore, the comprehensive characterization of the thickness helped determine that the device of 8.4 μm can still be used in all the visible spectrum since the diattenuation effect is not that strong with small thicknesses.

## Figures and Tables

**Table 1:** Δn values of LC E7 for 473 nm, 532 nm, and 633 nm at 25 °C.

λ(nm)	Δn
473	0.25
532	0.23
633	0.21

**Table 2:** Normalized amplitude absorption factors for 5.5 μm and 10 μm cells.

Device (Theoretical Thickness)	λ (nm)	Absorption Factors (Non-Illuminated Area)	Absorption Factors (Photoaligned Area)
Device 5.5 μm	633	$p_{x}$ = 0.94 ± 0.03	$p_{x}$ = 0.87 ± 0.03
		$p_{y}$ = 0.95 ± 0.03	$p_{y}$ = 0.91 ± 0.03
	532	$p_{x}$ = 0.81 ± 0.01	$p_{x}$ = 0.70 ± 0.01
		$p_{y}$ = 0.83 ± 0.01	$p_{y}$ = 0.84 ± 0.01
	473	$p_{x}$ = 0.76 ± 0.02	$p_{x}$ = 0.62 ± 0.02
		$p_{y}$ = 0.78 ± 0.02	$p_{y}$ = 0.79 ± 0.02
Device 10 μm	633	$p_{x}$ = 0.90 ± 0.02	$p_{x}$ = 0.83 ± 0.02
		$p_{y}$ = 0.92 ± 0.02	$p_{y}$ = 0.85 ± 0.02
	532	$p_{x}$ = 0.68 ± 0.02	$p_{x}$ = 0.35 ± 0.02
		$p_{y}$ = 0.70 ± 0.02	$p_{y}$ = 0.71 ± 0.02
	473	$p_{x}$ = 0.66 ± 0.01	$p_{x}$ = 0.18 ± 0.01
		$p_{y}$ = 0.67 ± 0.01	$p_{y}$ = 0.69 ± 0.01

**Table 3:** The average thickness value measured from the five points of the samples and for the two methods.

Silica Spacer Size (μm)	“Spectrometer” Thickness (μm)	“Polarimetric” Thickness (μm)
5.5 ± 0.4	8.5 ± 0.1	8.4 ± 0.2
10.0 ± 0.4	12.1 ± 0.1	12.0 ± 0.2

**Table 4:** Azimuth, ellipticity, and DOP of five points distributed along photoaligned area of the 8.4 μm thickness device for 0 V.

	Azimuth (°)	Ellipticity (°)	DOP (%)
Point 1	46.0	11.8	93.1
Point 2	47.1	10.5	93.4
Point 3	47.4	11.6	94.4
Point 4	45.2	12.1	94.3
Point 5	47.1	10.8	94.9

**Table 5:** Azimuth, ellipticity, and DOP for measurements in Figure 4 (central point).

Voltage (V)	Azimuth (°)	Ellipticity (°)	DOP (%)
1.1	−47.1	0.2	94.6
2.1	−88.7	41.4	93.4
2.6	48.5	0.1	92.1
3.0	48.3	12.4	92.7
4.3	87.2	43.6	95.0
10.0	45.8	17.7	99.0

**Table 6:** Azimuth, ellipticity, and DOP of five points distributed along photoaligned area of the 12.0 μm thickness device for 0 V.

	Azimuth (°)	Ellipticity (°)	DOP (%)
Point 1	45.4	14.6	97.0
Point 2	45.4	14.9	97.0
Point 3	44.9	12.6	97.1
Point 4	45.0	13.2	97.3
Point 5	45.6	14.5	97.0

**Table 7:** Azimuth, ellipticity, and DOP for measurements in Figure 5 (central point).

Voltage (V)	Azimuth (°)	Ellipticity (°)	DOP (%)
1.1	−47.4	0.2	97.6
1.5	88.9	44.0	99.2
1.7	45.9	0.1	98.9
3.1	41.8	17.0	99.0
6.5	−2.2	44.1	99.9
16.0	−45.5	18.1	100.0

## Data Availability

Data can be found in the figures in the manuscript.
